# Learning Descriptors Invariance through Equivalence Relations within Manifold: A New Approach to Expression Invariant 3D Face Recognition

**DOI:** 10.3390/jimaging6110112

**Published:** 2020-10-22

**Authors:** Faisal R. Al-Osaimi

**Affiliations:** Al-Abediah Campus, Umm Al-Qura Univeristy, Taif Rd., Makkah 21955, Saudi Arabia; frosaimi@uqu.edu.sa

**Keywords:** descriptor invariance, expression invariance, 3D face recognition, manifolds

## Abstract

This paper presents a unique approach for the dichotomy between useful and adverse variations of key-point descriptors, namely the identity and the expression variations in the descriptor (feature) space. The descriptors variations are learned from training examples. Based on labels of the training data, the equivalence relations among the descriptors are established. Both types of descriptor variations are represented by a graph embedded in the descriptor manifold. Invariant recognition is then conducted as a graph search problem. A heuristic graph search algorithm suitable for the recognition under this setup was devised. The proposed approach was tested on the FRGC v2.0, the Bosphorus and the 3D TEC datasets. It has shown to enhance the recognition performance, under expression variations, by considerable margins.

## 1. Introduction

3D face recognition has shown to achieve a considerable recognition accuracy and robustness, especially when compared to its 2D counterpart. There are vital applications of face recognition such as security, access control and human-machine interaction. The importance of its applications combined with the recent advances in the 3D digitization technologies have been the driving forces behind the interest in 3D face recognition among researchers. Despite reported advances in 3D face recognition in the recent years, the practical applications of 3D face recognition require even higher accuracies and robustness.

A particularly interesting recognition paradigm is concerned with the detection of key-points and then the extraction of descriptors from the local 3D surfaces around them. This paradigm inherently enjoys desirable properties such as the robustness to clutter and occlusions and the enabling of partial surface matching. While this paradigm has been very successful in the general object recognition [1,2,3], face recognition performance of the well-known approaches in this paradigm remained, until recently, below that of the state-of-the-art [4,5]. Recently, the rotation-invariant and adjustable integral kernel (RAIK) approach [6] which belongs to this paradigm has shown to highly perform in 3D face recognition when the matching is limited to the semi-rigid regions of the face (the nose and the forehead). The RAIK descriptors are discriminative, invariant to 3D rotations and completely representative of the underling surface. In the context of 3D face recognition, the descriptors also encode the expression variations. The 3D face variations pertaining to the identities of the individuals are essential for correct recognition. However, in practice these valuable variations mix with expression variations and rigid transformation, affecting the performance and robustness of face recognition.

When descriptors faithfully encode shape information of the local surfaces independently from the rigid transformations, it is imperative to assume that the local descriptors represent only the identity related shapes and the expression deformations of the local surfaces. One can consider expression deformations as *displacements* if descriptors, in the descriptor space, in an unknown and complex way. The differentiation between the two types of the descriptor displacements, namely the identity-induced displacements (IIDs) and the expression-induced displacements (EIDs), is learned and utilized to enhance recognition. The displacements between descriptors extracted from different scans of the same person and from the same location on the face (corresponding key-points) but under different facial expressions are basically EID. These displacements along with the displacements that set different individuals apart are extracted from training data and represented by a large graph embedded in the descriptor manifold for the utilization in the expression invariant face recognition. These displacements can be also perceived as one-to-one relations from the descriptor space to itself. In this terminology, the sub-graphs corresponding to the descriptors (the nodes) of the same individual under varying facial expressions represent equivalence relations (ERs) but the sub-graphs connecting the descriptors of different individuals represent identity relations (IRs).

**Figure 1:**
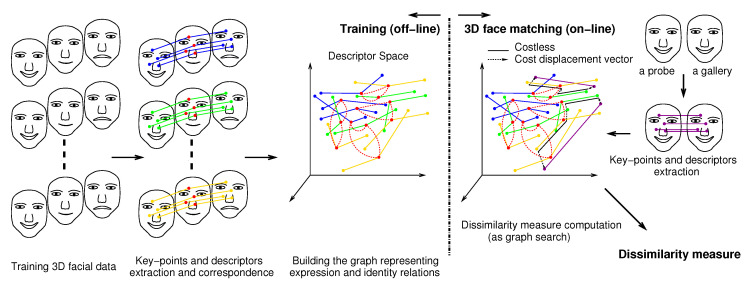
An Overview illustration of the proposed approach (better seen in color).

In the rest of the paper, the term *ensemble* refers to the set of descriptors extracted from a 3D facial scan. The set of ensembles that are extracted from multiple 3D facial scans of a person is called a *collection*. Many training collections of descriptors are extracted from sets of 3D facial scans of many individuals. The 3D facial scans in each collection are under varying expressions, including the neutral expression. The descriptors of each ensemble are mapped (linked) to their corresponding descriptors in the other ensembles. This results in multiple sets of corresponded descriptors within each collection. Each one of them is then joined together by a simple spanning graph which represents the equivalence relations, ERs. Between the different collections, the corresponding equivalence graphs (ERs) are connected to each other, enabling the representation of the identity relations, IRs. One viable approach is to inter-connect the different equivalence graphs from the descriptors of a neutral ensemble to the descriptors of neutral ensembles in other collections (neutral to neutral connections). For invariant matching of a probe ensemble to a gallery ensemble, the descriptors are first corresponded. Then, dissimilarity measures between descriptors are found by searching the graph for the path connecting each corresponded descriptor pair such that the encountered IRs along the graph path have cost (as a vector quantity), the encountered ERs have zero-cost and the magnitude of the sum of all the encountered IRs is minimum. Finally, on the basis of the minimized IR quantities the dissimilarity measures are computed. See Figure 1 for a graphical illustration.

## 2. The Literature Review

The approaches to expression invariant 3D face recognition can be broadly categorized into two categories; the image space approaches and the feature space approaches. Dealing with 3D surfaces in the image space is more intuitive. It is not surprising to find early successful approaches in this category. The avoidance of the highly deformable regions of the face such as the mouth and the cheeks, e.g., [7,8,9,10], has shown to be considerably effective. Unfortunately, the considered regions of the face still undergo expression deformations which can adversely affect the recognition accuracy. Another class of methods in the same category is to deform the 3D facial surface in an attempt to remove or at least reduce the effects of the expression deformations. The approach proposed in [11], transforms the 3D facial surface by bending the surface while preserving the geodesic distances among its points into an invariant image. As that approach flattens the expressions, it also flattens some other facial surface geometries. Recently, several methods extract expression invariant measures from geodesic distances of the facial surface [12,13,14]. The annotated face model, which is an elastic deformable model [15], deforms a 3D face under one expression to a target 3D face under another expression [16]. The recognition is then performed on the fitted model. It is not clear how such model can differentiate between the expression deformations and the other unique geometries of the face. In another work [17], a low dimensional principal component analysis (PCA) subspace in which the expression deformations reside is found from the 3D shape residues of registered pairs of non-neutral and neutral scans, where each pair belong to the same subject. For invariant recognition, the expression deformations in the image space are separated from novel 3D residues based on their projection onto the subspace. These methods are better situated for global face matching. Therefore, their robustness and accuracy may be undermined under occlusions and wide pose variations. The work in [18], performs elastic invariant matching of local surface regions around a handful of manually picked facial anatomical landmarks. Nonetheless, reliable automatic localization of the facial landmarks remains an open problem.

In a wide range of approaches, the matching is performed based on extracted features [19,20,21,22,23,24]. Feature extraction reduces the dimensionality of the facial data. An independent set of features is preferable for the recognition. Some features may be less sensitive to the expressions than others, providing a limited level of expression invariance. Typical examples of feature extraction methods are the linear subspace methods such as the different variants of PCA [25,26,27,28,29], linear discriminant analysis (LDA) [26,28] and independent component analysis (ICA) [30,31,32].

In the feature space, there are different methods in the literature that attempt to find the space regions that are associated with certain identities or class labels. One widely used methods is to estimate a probability density function (PDF) over space regions. The estimation of a PDF in a multidimensional vector space faces practical challenges. The number of training data samples required to compute such PDF grows exponentially with the number of dimensions, referred to as “the curse of dimensionality” [33,34]. Strong assumptions about the PDF are often made, such as assuming normal distribution [35,36], to survive on the available training samples. The “kernel trick” method was used in several approaches feature space approaches, e.g., [37,38,39]. The kernel function replaces the dot product in their non-kernelized variants, e.g., the support vector machines k-SVMs [39] and k-PCA [40], they are often data driven. Its overall process can be considered as a nonlinear transformation of the feature space to induce more separable classes. While it provides an elegant approach for the non-linear separability, it does not explicitly address the expression variations. In contrast, the proposed work is explicit in handling expression variations and versatile in selecting the relevant subset of the driving data for each match.

The manifold methods [41,42,43,44,45] in most cases are concerned with the non-linear dimensionality reduction, where the feature distribution in the feature space may be locally of a much lower dimension than that of the manifold as a whole. The de facto example is a “Swiss roll” surface residing in a higher dimensional space. Typically, rather than using the Euclidean distances for matching, the geodesic distances on the manifold are used instead [46]. Sometimes the problem at consideration or its formulation guarantees that the feature data form a manifold of a specific low dimension and the availability of enough feature samples to recover the lower dimensional manifold. An example of such a problem is the manifold of a rotated template image [47], the manifold dimension in this case is the number of the degree of freedom and the data samples can be generated as needed. The expression and identity manifold is of a complex structure with several dimensions (may vary locally). This problem also lacks the availability of enough data samples to accurately recover the manifold. In contrast, the proposed does not attempt to recover the lower dimension of the manifold, unroll it, or extract geodesic distances. It perceives the manifold as a sparse distribution of data samples and the distances between certain points are shortened to zero.

## 3. The Proposed Approach

This section describes the steps of the proposed approach and discusses the concepts behind them in more details than previously provided.

### 3.1. Conceptual Analysis

Manifold has the notion of the Euclidean spaces (tangential spaces) locally at each of its points. Conventionally, a descriptor space is either treated as one Euclidean space or as a manifold. In both situations, the large distances in the descriptor space translate into large dissimilarity measures which is not plausible for the recognition under variations. In contrast, the proposed approach has the ability to bring and merge distant tangential manifold spaces with each other. Consequently, the proximity and the non-proximity among the manifold points can combine and contribute more meaningfully to the dissimilarity measure. This can be achieved through the establishment of equivalence relations between reference points (corresponded descriptors) in the descriptor manifold. Let $Q=\{D_{1},\cdots ,D_{n}\}$ be a set of corresponded descriptors (an equivalence set) and $T_{D_{i}}M$ denotes a tangential space at the *i*-th descriptor of the manifold, *M*. Each corresponded pair of descriptors, $\left\{D_{i},D_{j}\right\}$, establishes an equivalence between all the tangential spaces in which $D_{i}$ and $D_{j}$ exist. The equivalency also extends to the manifold points around the descriptors. The same concept similarly applies for all the pair combinations of the descriptors in Q. This gives rise to the notion of the tangential space as quotient space, $Q_{Q}M$.

**Figure 2:**
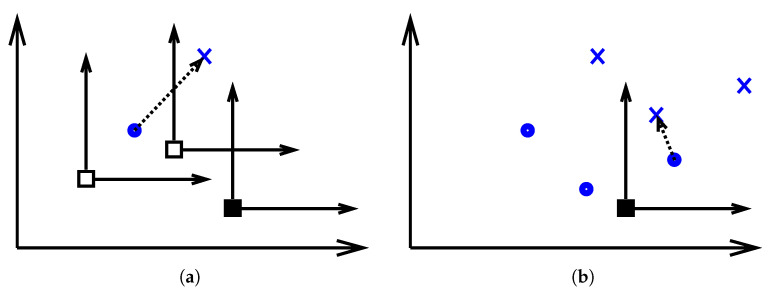
In (**a**), two manifold points appearing in the tangential spaces at three equivalent manifold points (the square ones). It shows the displacement vector between the two points. In (**b**), the original two points and their images under the equivalency are shown with respect to the reference tangential space (the solid square). The invariant displacement vector under the equivalency is also shown.

The displacement vectors and distances based on which the dissimilarity measures are computed can be computed in the quotient space. Let x and y be two manifold points and the displacement between them d(x,y) is to be computed under the equivalence set Q. The equivalent images of x and y in all the tangential spaces at the different Q points are mapped to a reference tangential space (the quotient space) which can be the tangential space at any Q point. The mapping from the *i*-th tangential space to the *r*-th tangential space, $E_{i}^{r}\left(x\right)$, is provided by Equation (1) and similarly the mapping $E_{j}^{r}\left(x\right)$ is provided by Equation (2). In the reference space there will be multiple images of x and y points and the displacement between them is defined as the displacement with the minimum magnitude (norm) between any equivalent image of *x* to any equivalent image of y, Equation (4), where ∥·∥ is the vector norm of the displacement vector and $t=(t_{1},t_{2},t_{3})$ is the optimal triple of equivalent descriptors. A graphical illustration is provided in Figure 2.

(1) $$\begin{array}{ccc} E_{i}^{r}\left(x\right) & = & x+D_{r}-D_{i}. \end{array}$$

(2) $$\begin{array}{ccc} E_{j}^{r}\left(y\right) & = & y+D_{r}-D_{j}. \end{array}$$

(3) $$\begin{array}{ccc} t & = & {\arg\min}_{\left(D_{i},D_{j},D_{r}\right)\subset Q^{3}}∥E_{i}^{r}\left(x\right)-E_{j}^{r}\left(y\right)∥. \end{array}$$

(4) $$\begin{array}{ccc} d(x,y) & = & E_{t_{1}}^{t_{3}}\left(x\right)-E_{t_{2}}^{t_{3}}\left(y\right). \end{array}$$

On the basis of the mapping relations, Equations (1) and (2), the differentiation between the expression variations and the identity variations can be achieved. The expression variation of a descriptor from one (the *i*-th) expression to another (the *j*-th) can be considered as the additive displacement vector $\Delta E_{i}^{j}=D_{j}-D_{i}$ and then separated from the identity variation. Therefore, $E_{i}^{j}\left(\cdot \right)$ modifies (or displaces) the expression of the input descriptor from expression *i* to expression *j*.

In the next discussions, the term “identity” refers to the person identity for collections and ensembles and to the space point (location) for the unexpressed descriptors. Suppose that the identity of the descriptor x is $p_{1}$ and the identity of the descriptor y is $p_{2}$. The descriptors x and y have both expression ($e_{x}$ and $e_{y}$) and identity (I(x) and I(y)) components, Equations (5) and (6). The identity variation $\Delta I_{p_{1}}^{p_{2}}\left(x,y\right)$ is defined as the difference between the two identity components, Equation (7).

(5) $$\begin{array}{ccc} x & = & I\left(x\right)+e_{x}. \end{array}$$

(6) $$\begin{array}{ccc} y & = & I\left(y\right)+e_{y}. \end{array}$$

(7) $$\begin{array}{ccc} \Delta I_{p_{1}}^{p_{2}}\left(x,y\right) & = & I(y)-I(x). \end{array}$$

The identity variation $\Delta I_{p_{1}}^{p_{2}}\left(x,y\right)$ can be computed based on the displacement d(x,y) (Equation (4)) as shown in Equations (11) and (12).

(8) $$\begin{array}{ccc} d(x,y) & = & E_{i}^{r}\left(x\right)-E_{j}^{r}\left(y\right), \end{array}$$

(9) $$\begin{array}{ccc} & = & x-y+D_{j}-D_{i}, \end{array}$$

(10) $$\begin{array}{ccc} & = & I\left(x\right)+e_{x}-I\left(y\right)-e_{y}+D_{j}-D_{i},\;\mathrm{and} \end{array}$$

(11) $$\begin{array}{ccc} & = & -\Delta I_{p_{1}}^{p_{2}}\left(x,y\right)-\Delta E_{x}^{i}\left(x\right)+\Delta E_{y}^{j}\left(y\right). \end{array}$$

When the expression of x is close (or ideally equals to) the expression $D_{i}$ and that of y equals to the expression $D_{j}$ then $\Delta I_{p_{1}}^{p_{2}}$ reduces to −d(x,y), Equation (12). This requirement is seamlessly achieved during the computation of d(x,y) according to Equation (4).

(12) $$\begin{array}{c} \Delta I_{p_{1}}^{p_{2}}\left(x,y\right)=-d\left(x,y\right). \end{array}$$

While the identity variations $\Delta I_{p_{1}}^{p_{2}}\left(x,y\right)$ is computed based on the descriptors x and y, $\Delta I_{p_{1}}^{p_{2}}\left(.,.\right)$ is independent of any particular descriptors as long they belong to the specified identities and at the corresponding key-points on the face. Therefore, the norm value of $\Delta I_{p_{1}}^{p_{2}}\left(x,y\right)$ or equally that of d(x,y) can be used in the computation of an invariant dissimilarity measure.

In general, the identity of a descriptor x can be changed from $p_{1}$ to the identity of any known descriptor, $I\left(.\right)=p_{k}$, as in Equation (13).

(13) $$\begin{array}{c} I_{p_{1}}^{p_{2}}\left(x\right)=x+\Delta I_{p_{1}}^{p_{k}}\Rightarrow I\left(I_{p_{1}}^{p_{k}}\left(x\right)\right)=p_{k} \end{array}$$

Similarly, the expression of a descriptor x can be changed from $e_{x}$ to that of any known descriptor, $E\left(.\right)=e_{k}$, as in Equation (14).

(14) $$\begin{array}{c} E_{e_{x}}^{e_{k}}\left(x\right)=x+\Delta E_{e_{x}}^{e_{k}}\Rightarrow E\left(E_{e_{x}}^{e_{k}}\left(x\right)\right)=e_{k} \end{array}$$

The identity and expression (changing) relations can be composed in any arbitrary sequence, e.g., that given in Equation (15).

(15) $$\begin{array}{ccc} x^{\prime } & = & E_{e_{1}}^{e_{2}}\circ I_{p_{2}}^{p_{3}}\circ E_{e_{x}}^{e_{1}}\circ I_{p_{1}}^{p_{2}}\left(x\right), \end{array}$$

(16) $$\begin{array}{ccc} & = & E_{e_{1}}^{e_{2}}\circ E_{e_{x}}^{e_{1}}\circ I_{p_{2}}^{p_{3}}\circ I_{p_{1}}^{p_{2}}\left(x\right),\mathrm{and} \end{array}$$

(17) $$\begin{array}{ccc} & = & E_{e_{x}}^{e_{2}}\circ I_{p_{1}}^{p_{3}}\left(x\right). \end{array}$$

(18) $$\begin{array}{ccc} E(x^{\prime }) & = & e_{2}. \end{array}$$

(19) $$\begin{array}{ccc} I(x^{\prime }) & = & p_{3}. \end{array}$$

The identity and expression relation composition provides the proposed approach with the capacity to utilize the training data in invariant recognition. Let x and y be two novel descriptors respectively with identities *p* and *g*. To compute the invariant dissimilarity measure between them for example using just one equivalence relation, one need to search the training data for a pair of descriptors belonging to the same person *c* (an equivalence relation pair $Q=\{D_{1},D_{2}\}$) that minimizes Equation (22). Using the optimal *Q*, the identity variation $\Delta I_{p}^{g}$ is given equally by any of the displacements in Equation (24). Equations (20) and (21) basically find the image of x under the equivalence relation *Q*. Equation (21) is computable from the descriptors values but Equation (20) only illustrates its identity and expression composition. The dissimilarity measure $∥\Delta I_{p}^{p}∥$ is ideally zero (practically minimal) if both x and y have the same identity or non-zero (practically non-minimal) otherwise. Note that the descriptors form a graph path from x to y through $D_{1}$ and $D_{2}$. More than one equivalence relation (belonging to different people) can be utilized in estimating $\Delta I_{p}^{g}$, the details are provided in Section 3.4.

(20) $$\begin{array}{ccc} x^{\prime } & = & E_{D_{2}}^{D_{1}}\circ I_{c}^{p}\circ E_{D_{1}}^{D_{2}}\circ E_{p}^{D_{1}}\circ I_{p}^{c}\left(x\right) \end{array}$$

(21) $$\begin{array}{ccc} & = & E_{D_{1}}^{D_{2}}\left(x\right)=x+D_{2}-D_{1} \end{array}$$

(22) $$\begin{array}{ccc} Q & = & \arg\min_{Q}\left|\right|x^{\prime }-y\left|\right| \end{array}$$

(23) $$\begin{array}{ccc} y & = & I_{p}^{g}\circ E_{D_{1}}^{D_{2}}\left(x\right) \end{array}$$

(24) $$\begin{array}{ccc} \Rightarrow \Delta I_{p}^{g} & = & y-E_{D_{1}}^{D_{2}}\left(x\right)=y-D_{2}+D_{1}-x \end{array}$$

### 3.2. The Correspondence of the Descriptors

The correspondence of the descriptors is important for the proposed approach, since it enables the tracking of their identity and expression variations. For a pair of descriptor ensembles, $N_{1}$ and $N_{2}$, the correspondence is defined as the mapping of each descriptor $D_{i}\in N_{1}$ to one descriptor $D_{j}\in N_{2}$, such that each pair $\left\{D_{i},D_{j}\right\}$ correspond to a particular spatial location on the face. Let the correspondence of $N_{1}$ and $N_{2}$ be denoted by the set $M_{1,2}$ for which Equation (25) holds.

(25) $$\begin{array}{c} M_{1,2}\subset \left\{\left\{D_{i},D_{j}\right\}\;\;|\;\;D_{i}\in N_{1}\wedge D_{j}\in N_{2}\right\}. \end{array}$$

The correspondence is established based on a customized variant of the random sample consensus (RANSAC) algorithm [48]. The dissimilarity of the descriptors and their locations are both utilized. The descriptors in the two ensembles are initially matched against each other based on the dissimilarity measure in Equation (26). The summation is performed over all the descriptors. The descriptors in $N_{1}$ are corresponded to those in $N_{2}$ with the minimum dissimilarity measures.

(26) $$\begin{array}{c} d\left(D_{i},D_{j}\right)=\sum ∥D_{i}-D_{j}∥. \end{array}$$

This will result in many correctly corresponded pairs but also will result in mis-correspondences. Let the key-point sets of the ensembles $N_{1}$ and $N_{2}$ be denoted respectively by $K_{1}$ and $K_{2}$. Each key-point k in $K_{1}$ or $K_{2}$ is a vector of the 3D point coordinates, $k={\left[x,y,z\right]}^{⊤}$. To correct the mis-correspondences, the 3D rigid transformations, both the rotation R and the translation t, that transform the key-points in $K_{1}$ to their corresponded ones in $K_{1}$ are first estimated using least square fitting. Then, the error of transformation (the Euclidean distance error), defined in Equation (28), is used to split the correspondences into inliers and outliers by comparison against a threshold value of the transformation error, $e_{th}$.

(27) $$\begin{array}{ccc} k_{i}^{\prime } & =Rk_{i}+t,\;\;\;\;\;\;\;\; & k_{i}\in K_{1}. \end{array}$$

(28) $$\begin{array}{ccc} e & =∥k_{i}^{\prime }-k_{j}∥,\;\;\;\;\;\;\; & k_{j}\in K_{2}. \end{array}$$

The rigid transformations are then recalculated base on the inlier correspondences and the process is iterated until the change in the norms of the new R and t diminishes. Using the converged values of R and t, the key-points in $K_{1}$ are transformed and then re-corresponded only to the $K_{2}$ key-points in their vicinity.

### 3.3. The Construction of the Embedded Graph

As previously mentioned, the graph is constructed from a training set of descriptors D organized in a set of ensembles N and a set collections C.

(29) $$\begin{array}{ccc} D & = & \{D_{1},\cdots ,D_{\left|D\right|}\}. \end{array}$$

(30) $$\begin{array}{ccc} N & = & \left\{N_{1},\cdots ,N_{\left|N\right|}\right\},\;\;\mathrm{where}\;\;\forall_{D\in N,\;N\in N}D\in D. \end{array}$$

(31) $$\begin{array}{ccc} C & = & \left\{C_{1},\cdots ,C_{\left|C\right|}\right\},\;\;\;\;\;\mathrm{where}\;\;\forall_{N\in C,\;C\in C}N\in N. \end{array}$$

The ERs are extracted from the correspondences within the individual collections, between pairs of ensembles, while the IRs are extracted from those between pairs of collections.

Initially, sub-graphs representing the ERs are sequentially extracted from each collection $C_{i}$ in C. To achieve that, a list of ensemble pair combinations within the collection $C_{i}$ is generated and then each generated ensemble pair is corresponded as described. The use of all possible combinations may be overly redundant, especially for large number of ensembles within the collection. This is because the correspondence problem is transitive. The minimum number of such pairs required for connected sub-graphs is $|C_{i}|-1$, where $|C_{i}|$ is the number of ensembles in the collection. However, for robustness a sufficient level of redundancy is maintained, since the redundancy could mitigate possible errors in key-point localization and detection under different facial expressions.

A disciplined approach for the maintenance of the required redundancy is based on the reduction of the hope count between the ensembles, when representing the ensembles as graph nodes and the pair combinations as graph edges. First, a minimal connected graph, $G_{i}=\left(V_{i},E_{i}\right)$, is found, e.g., by connecting each ensemble to the next. Then, iteratively the graph diameter, the largest path between any two ensembles, is computed and an edge (an ensemble pair) is introduced between the two ensembles until the graph diameter falls below some predefined threshold. The correspondences are then computed for the ensemble pairs adjacent to every edge in $E_{i}$ of the graph $G_{i}$ and then combined in one correspondence set $M_{C_{i}}$ for the collection $C_{i}$, as in Equation (32).

(32) $$\begin{array}{c} M_{C_{i}}=\underset{\forall_{e\in E_{i}}}{⋃}M_{j,k}\left(N_{j},N_{k}\right),\;\mathrm{where}\;\left\{N_{j},N_{k}\right\}=e. \end{array}$$

The pairs in the set $M_{C_{i}}$, when each is considered as a graph edge, form a new graph $G_{C_{i}}$ with multiple connected sub-graphs $G_{C_{i},s}$, where $s=1,\cdots ,S_{i}$.

(33) $$\begin{array}{ccc} G_{C_{i}} & = & (V_{C_{i}},E_{C_{i}}). \end{array}$$

(34) $$\begin{array}{ccc} G_{C_{i},s} & = & \left(V_{C_{i},s},E_{C_{i},s}\right),\;\;V_{C_{i},s}\subset V_{C_{i}}\wedge E_{C_{i},s}\subset E_{C_{i}}. \end{array}$$

The number of the connected sub-graphs $S_{i}$ ideally should equal to the number of the descriptors per ensemble and the number of vertices of each subgraph $|V_{C_{i},s}|$ should equal to the number of the ensembles, $|C_{i}|$, in the collection. However, this does not necessarily hold in practice. In fact, the number of the vertices can be less than $|C_{i}|$ for some sub-graphs, due to possible failure to detect some key-points, or it can be particularly much higher than $|C_{i}|$, since more than one sub-graph can be joined together due to mis-correspondences. When the number of the vertices of any sub-graph is significantly low, it can be considered as an indication that the underling key-point is not repeatable and the sub-graph is discarded.

The larger connected sub-graphs are iteratively segmented (partitioned) based on the well-known spectral graph partitioning (clustering) algorithm [49,50]. The edges of the sub-graph to be partitioned are assigned the weight values shown in Equation (36). $w_{e_{l}}$ is the weight of the edge $e_{l}$ after normalization (by division by the average non-normalized weight $\bar{w}$). The distance d between the adjacent descriptors to the edge is the same defined in Equation (26).

(35) $$\begin{array}{ccc} w_{e_{l}}^{\prime }=\frac{1}{d(D_{j},D_{k})},\; & & \mathrm{where}\;\left\{D_{j},D_{k}\right\}=e_{l},\\ & & \mathrm{for}\;l=1,\cdots ,|E_{C_{i}},s|. \end{array}$$

(36) $$\begin{array}{ccc} w_{e_{l}}=\frac{w_{e_{l}}^{\prime }}{\bar{w}},\;\;\;\;\;\;\;\;\;\;\;\; & & \mathrm{for}\;l=1,\cdots ,|E_{C_{i}},s|. \end{array}$$

The Laplacian matrix $L_{C_{i},s}$ of the weighted and connected sub-graph is then computed.

(37) $$\begin{array}{c} L_{C_{i},s}=D_{C_{i},s}-A_{C_{i},s}. \end{array}$$

The diagonal matrix $D_{C_{i},s}$ is the degree matrix of the weighted graph, each diagonal element is the sum of the normalized weights of all the incident edges to the corresponding vertex of the graph. $A_{C_{i},s}$ is the adjacency matrix of the graph, each $a_{i,j}$ element is the normalized weight of the edge connecting the *i*-th vertex to the *j*-th vertex. The second smallest eigenvalue $\lambda_{f}$ of $L_{C_{i},s}$ is an indicator of how the graph is well-connected.

(38) $$\begin{array}{c} L_{C_{i},s}x_{f}=\lambda_{f}x_{f}. \end{array}$$

The corresponding eigenvector to $\lambda_{f}$ is known as the Fiedler vector, $x_{f}$. The Fielder vector elements has comparable values for strongly connected vertices. In the next step, the K-means clustering algorithm (with only two clusters) is applied to the elements of $x_{f}$. Based on the resulting two clusters of vertices, the graph $G_{C_{i},s}$ partitioned. The described graph partitioning is iteratively applied until, the resulting graphs are strongly connected and roughly of the expected number of vertices, $|C_{i}|$.

Finally, the vertices (the descriptors) of the resulting connected graphs are considered the equivalence sets $Q_{C_{i},s}$, where $s=1,\cdots ,S_{i}$, of the collection $C_{i}$. Each graph $G_{C_{i},s}$ is then simplified to the star graph $T_{C_{i},s}$ (the spanning graph). Every vertex descriptor in the equivalence set is connected to one neutral descriptor which is chosen as the nearest to the means of $Q_{C_{i},s}$ in case there are multiple neutral descriptors, called the bridging vertex (or descriptor), denoted by $B_{i,s}$. Similarly, the equivalence sets and the equivalence star graphs are extracted for every collection. The equivalence graphs are then allowed to join corresponding ones in all other collections, from bridging to bridging vertices. These joining edges represent the IRs. It is possible not to define any particular bridging points and allow for IRs connections (edges) from all the equivalent vertices of one collection to all the vertices of the corresponding sets in all other collections. In this case, the spanning graph of the equivalence sets will be a line connecting them, rather than a star. However, the definition of the bridging points significantly simplifies the graph search problem (the matching), as will be discussed in the next subsection.

### 3.4. The Heuristic Graph Search

The graph search takes place during the matching of a probe ensemble $N_{p}$ to a gallery ensemble $N_{g}$. The two ensembles are first corresponded. Theoretically, an invariant dissimilarity measure can be computed between $N_{p}$ and $N_{g}$ for an optimal graph path $P^{*}\in P$ connecting them. In the general case, possible candidate paths start with $N_{p}$ then zero or more collections are visited and finally terminate at $N_{g}$. The paths should be simple, have no repeated edges or collections. At each visited collection two ensembles are visited (an entrance and an exit one). At the descriptor level, there are multiple paths (a path bundle) connecting the corresponded descriptors in parallel for each higher level path. The paths are eventually realized as sequences of descriptors. The entrance and exit ensembles may be fixed for the descriptor level path bundle. However, relaxing this requirement and letting the entrance and the exit ensembles to vary for the different descriptor level paths is beneficial when the variety of the training expressions in the collections are limited. Below are the definitions of the paths at the different levels.

(39) $$\begin{array}{cc} P^{\prime } & =\{\left\{N_{p},C_{I(1)},\ldots ,C_{I(|P|-2)},N_{g}\right\}\;|\;C_{I(\cdot )}\in C\}.\\ \; & \equiv \{\left\{N_{p},N_{I(1),E(1)},N_{I(1),X(1)},\ldots ,N_{I(|P|-2),E(|P|-2)},N_{I(|P|-2),X(|P|-2)},N_{g}\right\}\;|\;\\ & N_{I(\cdot ),E(.)}\in N\wedge N_{I(\cdot ),X(\cdot )}\in N\}.\\ \; & \equiv \{\left\{D_{p,i},D_{I(1),E(1)},D_{I(1),X(1)},\ldots ,D_{I(|P|-2),E(|P|-2)},D_{I(|P|-2),X(|P|-2)},D_{g,j}\right\} \end{array}$$

(40) $$\begin{array}{cc} & \;\;|\;D_{I(\cdot ),E(.)}\in D\wedge D_{I(\cdot ),X(.)}\in D\wedge \left\{D_{p,i},D_{g,j}\right\}\in M_{p,g}\}=P\cdot \end{array}$$

The multiple subscript indices of the path vertices uniquely point to the specific graph vertices and also indicate their identity I(·), expression whether it is the entrance expression E(·) and the exit expression X(·). Equation (41) shows how the invariant measure can be computed based on the graph paths.

(41) $$\begin{array}{cc} s^{\prime }= & \min_{P\in P}\sum_{\left\{x,y\right\}\in M_{p,g}}∥E_{E(|P|-2)}^{X(|P|-2)}\circ \cdots \circ E_{E(2)}^{X(2)}\circ E_{E(1)}^{X(1)}\left(x\right)-y∥. \end{array}$$

Existing optimal path searching algorithms such the Dijkstra and Bellman-Ford are not suitable for the solution of Equation (41). They deal scalar edge weights. In contrast, the edge weights in the tackled problem here are multidimensional vectors and the optimized quantity is the norm of their summation.

By considering only the bridging points, which were described earlier in Section 3.3, as the entrance and the exit vertices between collections, the graph density reduces and the maximum number of collections per path also reduces to two. This means that each considered path has at maximum three identity changes and two removable expressions. Apart from the constraint on the maximum path length, |P|, Equation (41) holds for this lighter version of the graph search problem.

The proposed heuristic graph search proceeds by assigning one collection to the probe ensemble and one collection to the gallery ensemble (possibly another one). These assignments are initially performed based on the vicinity (in the descriptor space) of the descriptors of the probe and the gallery ensembles to those in the assigned collections. A KD-tree of all the training descriptors was built during an off-line stage to enable an efficient search for the nearest neighbors. A table of the descriptors information containing their ensemble, collection and equivalence sets is associated with the KD-tree. The *k* nearest neighbors of each descriptor in the probe or the gallery ensemble vote for the different collections based on their associated information (labels). The collection that receives the highest number of votes is assigned to the ensemble. Next, the descriptors of $N_{p}$ and $N_{g}$ are assigned entrance and exit descriptors within the assigned collections. For this task, a separate KD-tree per collection was built (during an off-line stage), since smaller KD-tree are more efficient to search. Then, for each corresponded descriptor pair, $\left\{x,y\right\}\in M_{p,g}$ where $x\in N_{p}$ and $y\in N_{g}$, the nearest three descriptors to x are considered as potential entrance descriptors to the collection assigned to $N_{p}$. Similarly, the three nearest neighbors to y are found and considered as potential exit descriptors from the collection assigned to $N_{g}$. Among the few combinations of potential entrance and exit descriptors, the one which yields the lowest value of the scalar function $m^{\prime }\left(x,y\right)$, defined in Equation (42), is assigned to x and y as respectively their entrance and exit descriptors.

(42) $$\begin{array}{cc} m^{\prime }\left(x,y\right)= & ∥E_{E(2)}^{X(2)}\circ E_{E(1)}^{X(1)}\left(x\right)-y∥ \end{array}$$

(43) $$\begin{array}{cc} = & ∥\Delta I_{p}^{g}∥ \end{array}$$

At this point of time, only a good initial guess of the solution is found and the search for the optimal path (or measure) is not performed yet. Nonetheless, the most similar people (collections) and expressions are likely to be assigned to the probe and the gallery.

The optimization is carried out implicitly as nearest neighbor search. The descriptors of $N_{p}$ are first displaced as in Equation (44) which produces new images of descriptors, $x_{i}^{\prime }$ for $i=1,\cdots ,|N_{p}|$. The new descriptors are then used to re-assign the gallery a new training collection and new entrance and exit descriptors as described earlier. It is then followed by a similar displacement and re-assignment of the probe descriptors based on the images of the gallery descriptors, $y_{i}^{\prime }$ as in Equation (45). Each of the two steps implies the minimization of Equation (42).

(44) $$\begin{array}{c} x_{i}^{\prime }=E_{E(2)}^{X(2)}\circ E_{E(1)}^{X(1)}\left(x\right)=x+D_{I(1),X(1)}-D_{I(1),E(1)}+D_{I(1),X(2)}-D_{I(1),E(2)} \end{array}$$

(45) $$\begin{array}{c} y_{i}^{\prime }=E_{X(2)}^{E(2)}\circ E_{X(1)}^{E(1)}\left(y\right)=y+D_{I(1),E(1)}-D_{I(1),X(1)}+D_{I(1),E(2)}-D_{I(1),X(2)} \end{array}$$

This process is then iterated a few times until convergence. These steps are only committed when they result in further minimization of Equation (42).

The described graph search accounts for paths with one and two collections (as both the probe and the gallery can be assigned to the same collection). The direct path between the probe and the gallery should also be considered which is accounted for by the simple minimization in Equation (46).

(46) $$\begin{array}{c} m\left(x,y\right)=\min\{m^{\prime }\left(x,y\right),∥x-y∥\}. \end{array}$$

### 3.5. The Dissimilarity Measure between Ensembles

An overall dissimilarity measure, *s*, between any two ensembles can be computed from the dissimilarity measures between the corresponded descriptor pairs, i.e., the *m* values shown in Equation (46). The *N* descriptor measures with the least values are simply summed to produce an overall ensemble measure *s*, as in Equation (47). *N* is much less than the typical number of the corresponded descriptor pairs. This would avoid the measures with high values. For those ones the expression variations may not be effectively removed by the proposed approach. When computing the dissimilarity matrix, its entries are further normalized to range from zero to one for each probe.

(47) $$\begin{array}{c} s\left(N_{p},N_{g}\right)=\sum_{i}^{N}m_{i}. \end{array}$$

## 4. Experiments

A number of face recognition experiments were conducted on the FRGC v2.0 [51], the 3D TEC [52] and the Bosphorus [53] datasets. These datasets differ from each other in a number of aspects. First, the FRGC dataset has the largest number of individuals (466 people in the testing partition) among the three datasets. It has diverse facial expressions but about half of its facial scans are under neutral or near neutral expressions. On the other hand, the 3D TEC and the Bosphorus datasets can even pose a more significant challenge to the recognition under facial expression variations. In the case of the 3D TEC, the challenge mainly arises because the individuals are identical twins (107 twins/214 individuals). In the third and the fourth 3D TEC experiments, the probes and the galleries are under different facial expressions. In contrast, the probe and the gallery scans specified in the dataset for the first and the second 3D TEC experiments involve no expression variations. In the case of the Bosphorus dataset, there are many scans for only 105 different people. However, the facial expression challenge arises because the facial expressions are generally of a larger extent in comparison to the other two datasets.

### 4.1. RAIK Descriptor Based Experiments

As the proposed expression invariant approach requires a set of training data with many individuals and under different facial expressions including the neutral expression, the FRGC dataset is an appropriate choice for training the system. The FRGC dataset has a training partition. However, it has a limited number individuals and the individual sets of the training and the testing partitions are not mutually exclusive. For this reason, a significant part of the testing partition of the FRGC dataset was used for training the proposed system, all the facial scans of the first 300 individuals. The remaining scans of the testing partition was used for testing the proposed approach. A gallery of 166 neutral facial scans (one scan per subject) was formed. The remaining scans were split into a neutral and a non-neutral probe subsets. The trained system was then used to perform the tests on the 3D TEC and the Bosphorus datasets. In all the experiments including both the expression-invariant approach and the plain RAIK approach which was used for result comparison, the RAIK features were compressed using the principal component analysis (PCA), each to a vector of twenty PCA coefficients. The RAIK descriptor has two adjustable parameters α and β. They were respectively set to −0.15 and 0.0 for all the conducted experiments.

**Figure 3:**
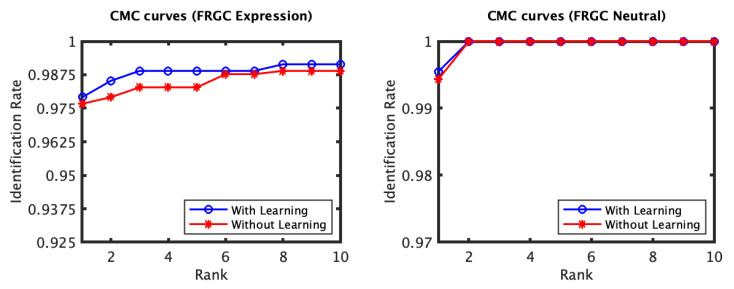
The CMC curves of the non-neutral (**left**) and the neutral (**right**) subsets of the FRGC dataset which were spared for evaluation. The curves compare the performance when using the proposed learning approach based on the RAIK features to that when the RAIK features are used without learning. Both methods achieve a very high performance. However, a noticeable improvement is observed for the non-neutral scans in particular, especially for the first few ranks.

**Figure 4:**
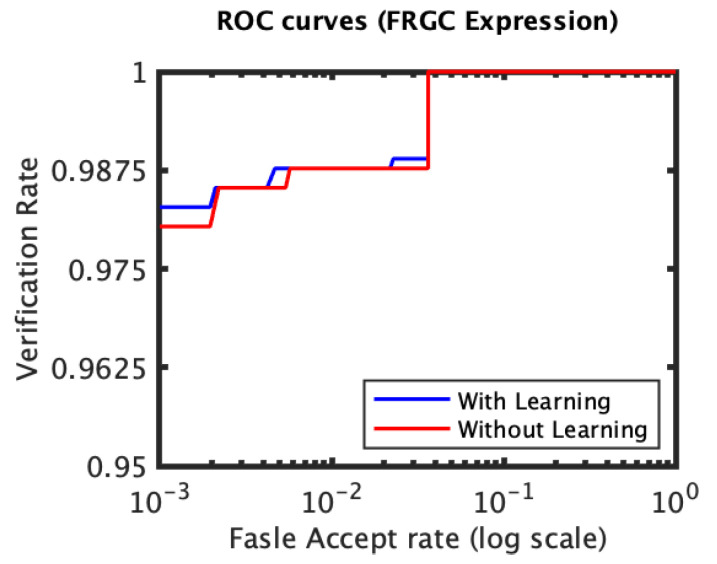
The ROC curves for the non-neutral subset of the FRGC dataset which were spared for evaluation (RAIK descriptor).

The recognition performance results of the experiments conducted on the FRGC dataset, Figure 3 and Figure 4, indicate that the proposed expression-invariant approach noticeably enhances the identification rates of the non-neutral probes at the first few ranks where the expression variations have more impact on the identification performance. While the first rank identification rate has increased from 97.69% (for the plain RAIK approach) to 97.90% (for the proposed approach based on the RAIK descriptors), the margin between the two rates has further increased at the second rank and peaked at the third rank where the identification rate has increased from 98.32% to 98.95%. It should be noted that the plain RAIK approach already achieves a very high identification performance because it limits the matching to the semi-rigid regions (the forehead and the nose) of the face. As more regions are considered, the identification rate margin between the proposed approach and the plain RAIK approach increases in favor of the proposed approach. This is because the identification rates of the plain RAIK approach declines more rapidly with the inclusion of the non-rigid regions of the face while proposed approach still declines but a slower pace. However, the performance of both approaches is optimal when only the semi-rigid regions of the face are considered. It could be concluded that the proposed approach contributes the reliability of the recognition in addition to the observed recognition performance enhancement. For the neutral experiment, the impact of the proposed approach is limited, which is expected as there are no expression variations and the identification rates for the neutral scans are already above 99.5%. Some verification rate improvement was observed for the non-neutral experiment but it was not significant. It has increased from 98.11 to 98.31% at 0.001 FAR.

**Figure 5:**
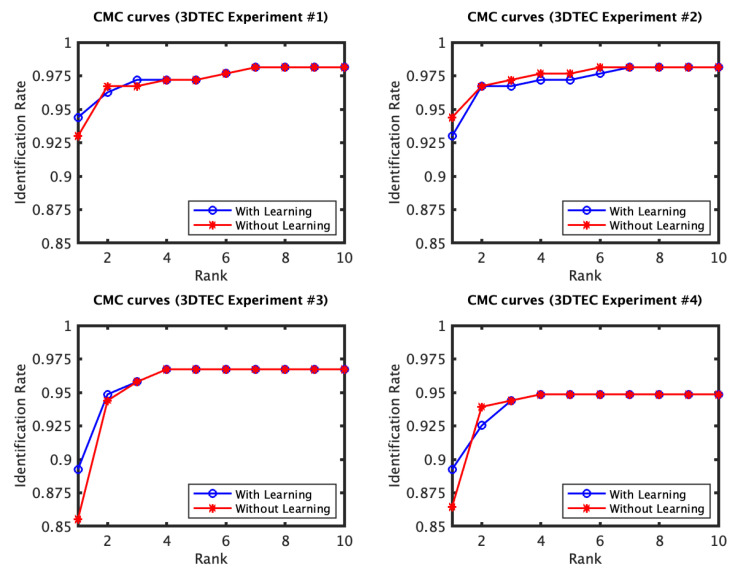
The CMC curves of the four experiments of the 3D TEC dataset performed using RAIK descriptor. The first two experiments involve no expression variations. For these two experiments there is a limited effect of the learning approach on the identification performance. In contrast, the third and fourth experiments have expression variations among the twins facial scans, for which the proposed approach has shown to considerably improve the recognition performance.

**Figure 6:**
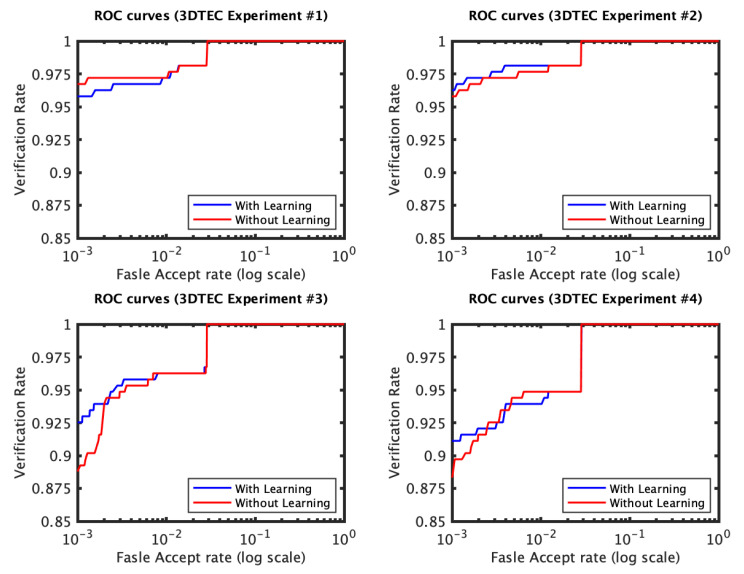
The ROC curves of the four 3D TEC experiments based on RAIK.

**Figure 7:**
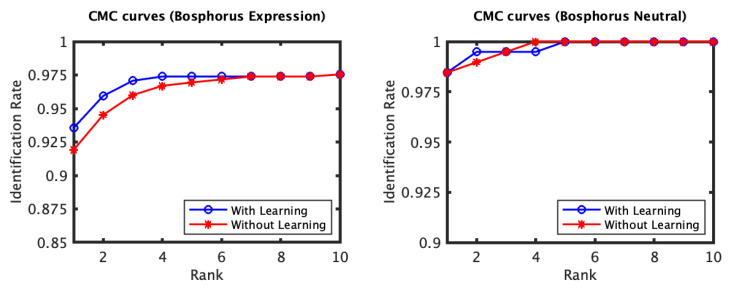
The CMC curves of the Bosphorus dataset (RAIK descriptor).

**Figure 8:**
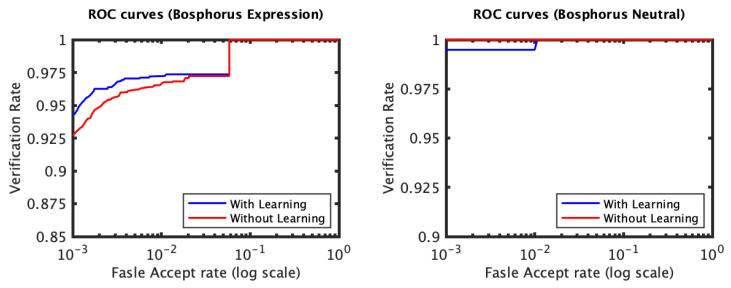
The ROC curves of the Bosphorus dataset (RAIK descriptor).

The identification and verification rates of the first and the second experiments of the 3D TEC dataset were not significantly impacted by the proposed approach. The interpretation of this observation is that for these two experiments the probe and the gallery scans of the twins are under the same expressions. In contrast, the impact of the proposed approach on the third and the fourth experiments was more significant (Figure 5 and Figure 6). The proposed approach has increased the first rank identification rate of the third experiment from 85.51 to 89.25% and from 86.45 to 89.25% for the fourth experiment. It appears from the 3D TEC and the FRGC results that the recognition enhancement of the proposed approach becomes more significant when the expression variations are more challenging to the plain RAIK approach. For these two experiments, the verification rates were respectively 92.52% and 91.12% at 0.001 FAR for the proposed approach, in comparison to 88.79% and 88.32% at 0.001 FAR for the plain RAIK approach. The results of the Bosphorus dataset indicate that the proposed approach enhances the recognition performance for the probes under non-neutral facial expressions (Figure 7 and Figure 8). The identification rate had increased from 91.94 to 93.55% and the verification rate had increased from 92.60 to 94.21% at 0.001 FAR. There was a negligible degradation in the verification performance of the neutral expression scans. Nonetheless, both the proposed system and the plain RAIK system had achieved above 99.5% verification at 0.001 FAR for the neutral scans.

### 4.2. Local 3D PCA Descriptor Based Experiments

The experiments in this subsection were conducted in a similar fashion to those performed using RAIK descriptor on the FRGC and the 3D TEC datasets. The RAIK descriptors were used instead of the local 3D PCA ones. Local 3D PCA descriptor is known to perform very well in 3D face recognition [4,54]. However, it is not rotation-invariant in contrast to RAIK. Therefore, the 3D faces were ensured to have a standard frontal pose using the principal components of the 3D face. The nose tip of the face was detected in the 3D face point-cloud and then the facial 3D points within 80 mm radius were cropped. They are then pose-corrected to their principal vertical and horizontal components and converted to range images. The local 3D PCA descriptors were extracted from 3D surface patches of 15 × 15 mm size. They are then translated along the *z*-coordinate so their central points have a depth value of zero. Finally, the dimensionality of the 3D local surfaces are reduced using standard PCA to 20D vectors.

3D face recognition using the proposed approach based on local PCA descriptors was performed and compared against the results of local PCA descriptors alone. The use of the proposed approach has shown to boost both the identification and verification rates of standard PCA when performed in similar settings. The improvement margins generally seem better than those observed for RAIK. However, the performance of the proposed approach based on RAIK is much better than the PCA one. This is probably because RAIK is rotation-invariant and more discriminative.

**Figure 9:**
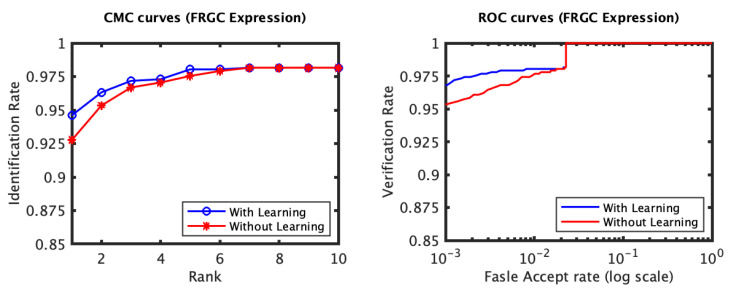
The CMC and ROC curves of the FRGC facial scans under expression using local 3D PCA descriptor.

**Figure 10:**
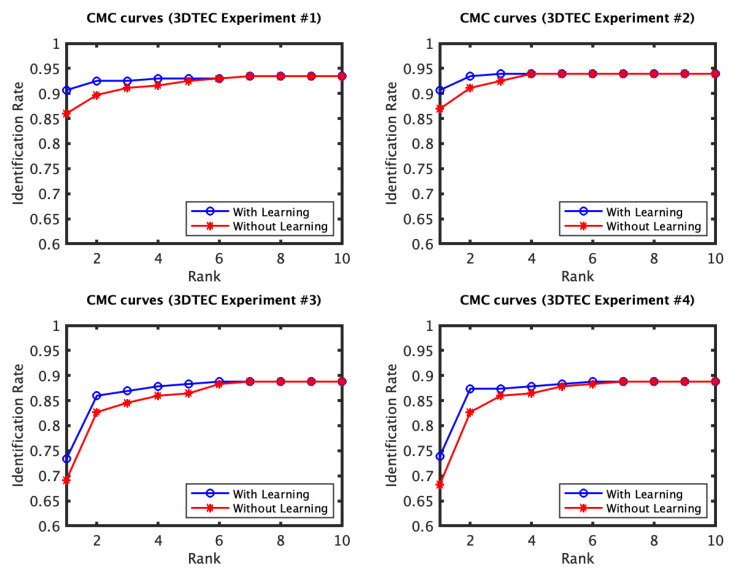
The CMC curves of the four 3D TEC experiments based on local 3D PCA descriptor.

**Figure 11:**
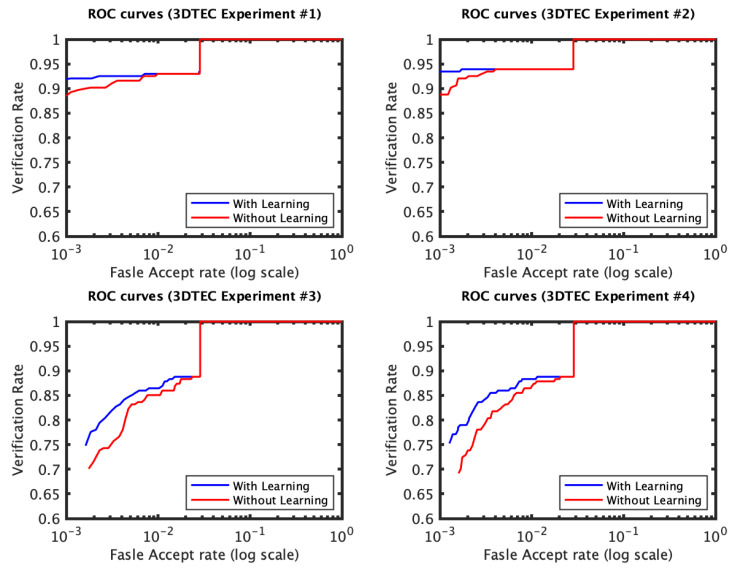
The ROC curves of the four 3D TEC experiments based on local 3D PCA.

The proposed approach based on local PCA has shown to improve rank-1 identification rate from 92.76 to 94.6% for the FRGC 3D faces under facial expressions, see Figure 9. The verification rate has also improved from 95.34 to 96.81% at 0.001 FAR. The performance on 3D TEC dataset has also experienced considerable improvements, see Figure 10 and Figure 11. The first rank identification rates when using the proposed approach were 90.65%, 90.65%, 73.36% and 73.83%, respectively for the four standard 3D TEC experiments (experiment-I, II, III and IV). Without the application of the proposed approach, the rates were respectively 85.98%, 86.92%, 69.16% and 68.22%. The verification rates at 0.001 respectively before and after the application of the proposed approach were 87.85% and 92.06% for the first experiment but 88.79% and 93.46% for the second one. The rates have also improved for the third and fourth experiments respectively from 70.09 to 74.77% and from 69.16 to 75.23% at roughly 0.0011 FAR (the lowest possible one).

In comparison to the recent 3D face recognition literature, the proposed approach achieves comparable results to the state-of-art even if it does not generally outperform recent deep learning based advances, especially those based on holistic face recognition [55,56]. However, the RAIK descriptor in particular already achieves a high recognition performance on its own and the proposed approach has shown to further improve its performance. Some of the results of the proposed approach discussed earlier are in close proximity or even exceed those of deep learning. For example, identification rate (89.25%) of the proposed approach using RAIK for the third and fourth 3D TEC experiments exceed those of the highly performing “deep 3D face identification” method described in [55] (whose rates are respectively 81.3% and 79.9%), see Table 1. Apart from recognition performance, conducting 3D face recognition based on local features may have advantages over holistic face recognition since it does not require the visibility of the whole face for matching.

## 5. Conclusions

The proposed research described a new manifold-based approach for learning facial expression invariance of the key-point descriptors. The descriptor variations induced by the facial expressions were handled with the equivalence relations within the descriptor manifold. Then, invariant dissimilarity measures (distances) were computed based on the equivalence relations. This approach has shown to improve the recognition performance especially when the facial scans being matched involve expression variations.

## Figures and Tables

**Table 1:** Rank-1 identification rate comparisons on the three datasets.

Approach	FRGC V2.0		Bosphorus	3D TEC			
	Expression	Neutral	(Expression)	Exp. I	Exp. II	Exp. III	Exp. IV
Li et al. [57]	96.3% (combined)		98.82%	NA	NA	NA	NA
Kim et al. [55]	NA	NA	99.2%	94.8%	94.8%	81.3%	79.9%
This paper	97.90%	99.55%	93.55%	94.39%	92.99%	89.25%	89.25%

## References

[1] Lowe D.G. (1999). Object recognition from local scale-invariant features. Proceedings of the Seventh IEEE International Conference on Computer Vision.

[2] Belongie S., Malik J., Puzicha J. (2002). Shape matching and object recognition using shape contexts. IEEE Trans. Pattern Anal. Mach. Intell..

[3] Rublee E., Rabaud V., Konolige K., Bradski G. ORB: An efficient alternative to SIFT or SURF. Proceedings of the 2011 IEEE International Conference on Computer Vision (ICCV).

[4] Mian A.S., Bennamoun M., Owens R. (2008). Keypoint detection and local feature matching for textured 3D face recognition. Int. J. Comput. Vis..

[5] Al-Osaimi F., Bennamoun M., Mian A. Interest-point based face recognition from range images. Proceedings of the British Machine Vision Conference.

[6] Al-Osaimi F.R. (2015). A novel multi-purpose matching representation of local 3D surfaces: A rotationally invariant, efficient, and highly discriminative approach with an adjustable sensitivity. IEEE Trans. Image Process..

[7] Mian A.S., Bennamoun M., Owens R. (2007). An efficient multimodal 2D-3D hybrid approach to automatic face recognition. IEEE Trans. Pattern Anal. Mach. Intell..

[8] Chang K.J., Bowyer K.W., Flynn P.J. Effects on facial expression in 3D face recognition. Proceedings of the Defense and Security, International Society for Optics and Photonics.

[9] Chang K.I., Bowyer W., Flynn P.J. (2006). Multiple nose region matching for 3D face recognition under varying facial expression. IEEE Trans. Pattern Anal. Mach. Intell..

[10] Faltemier T.C., Bowyer K.W., Flynn P.J. (2008). A region ensemble for 3-D face recognition. IEEE Trans. Inf. Forensics Secur..

[11] Bronstein A.M., Bronstein M.M., Kimmel R. (2003). Expression-invariant 3D face recognition. Proceedings of the Audio-and Video-Based Biometric Person Authentication.

[12] Drira H., Ben Amor B., Srivastava A., Daoudi M., Slama R. (2013). 3D face recognition under expressions, occlusions, and pose variations. IEEE Trans. Pattern Anal. Mach. Intell..

[13] Smeets D., Hermans J., Vandermeulen D., Suetens P. (2012). Isometric deformation invariant 3D shape recognition. Pattern Recognit..

[14] Berretti S., Del Bimbo A., Pala P. (2010). 3D face recognition using isogeodesic stripes. IEEE Trans. Pattern Anal. Mach. Intell..

[15] Metaxas D.N., Kakadiaris I.A. (2002). Elastically adaptive deformable models. IEEE Trans. Pattern Anal. Mach. Intell..

[16] Murtuza M.N., Lu Y., Karampatziakis N., Theoharis T. (2007). Three-Dimensional Face Recognition in the Presence of Facial Expressions: An Annotated Deformable Model Approach. IEEE Trans. Pattern Anal. Mach. Intell..

[17] Al-Osaimi F., Bennamoun M., Mian A. (2009). An expression deformation approach to non-rigid 3D face recognition. Int. J. Comput. Vis..

[18] Maalej A., Amor B.B., Daoudi M., Srivastava A., Berretti S. (2011). Shape analysis of local facial patches for 3D facial expression recognition. Pattern Recognit..

[19] Gökberk B., İrfanoğlu M.O., Akarun L. (2006). 3D shape-based face representation and feature extraction for face recognition. Image Vis. Comput..

[20] Lu X., Jain A.K. (2005). Multimodal Facial Feature Extraction for Automatic 3D Face Recognition.

[21] Xu C., Li S., Tan T., Quan L. (2009). Automatic 3D face recognition from depth and intensity Gabor features. Pattern Recognit..

[22] Tan X., Triggs B. (2007). Fusing Gabor and LBP feature sets for kernel-based face recognition. Analysis and Modeling of Faces and Gestures.

[23] Wang Y., Liu J., Tang X. (2010). Robust 3D face recognition by local shape difference boosting. IEEE Trans. Pattern Anal. Mach. Intell..

[24] Singh C., Mittal N., Walia E. (2011). Face recognition using Zernike and complex Zernike moment features. Pattern Recognit. Image Anal..

[25] Turk M., Pentland A. (1991). Eigenfaces for recognition. J. Cogn. Neurosci..

[26] Belhumeur P.N., Hespanha J.P., Kriegman D. (1997). Eigenfaces vs. fisherfaces: Recognition using class specific linear projection. IEEE Trans. Pattern Anal. Mach. Intell..

[27] Blanz V., Vetter T. (2003). Face recognition based on fitting a 3D morphable model. IEEE Trans. Pattern Anal. Mach. Intell..

[28] Wang X., Tang X. (2004). A unified framework for subspace face recognition. IEEE Trans. Pattern Anal. Mach. Intell..

[29] Russ T., Boehnen C., Peters T. 3d face recognition using 3d alignment for pca. Proceedings of the 2006 IEEE Computer Society Conference on Computer Vision and Pattern Recognition.

[30] Draper B.A., Baek K., Bartlett M.S., Beveridge J.R. (2003). Recognizing faces with PCA and ICA. Comput. Vis. Image Underst..

[31] Harguess J., Aggarwal J. A case for the average-half-face in 2D and 3D for face recognition. Proceedings of the 2009 IEEE Computer Society Conference on Computer Vision and Pattern Recognition Workshops.

[32] Liu C., Wechsler H. (2003). Independent component analysis of Gabor features for face recognition. IEEE Trans. Neural Netw..

[33] Verleysen M., François D. (2005). The curse of dimensionality in data mining and time series prediction. Computational Intelligence and Bioinspired Systems.

[34] Jain A., Zongker D. (1997). Feature selection: Evaluation, application, and small sample performance. IEEE Trans. Pattern Anal. Mach. Intell..

[35] Paalanen P., Kamarainen J.K., Ilonen J., Kälviäinen H. (2006). Feature representation and discrimination based on Gaussian mixture model probability densities—Practices and algorithms. Pattern Recognit..

[36] Raudys S.J., Jain A.K. (1991). Small sample size effects in statistical pattern recognition: Recommendations for practitioners. IEEE Trans. Pattern Anal. Mach. Intell..

[37] Schölkopf B., Smola A., Müller K.R. (1998). Nonlinear component analysis as a kernel eigenvalue problem. Neural Comput..

[38] Hotta K. (2008). Robust face recognition under partial occlusion based on support vector machine with local Gaussian summation kernel. Image Vis. Comput..

[39] Ksantini R., Boufama B.S., Ahmad I.S. (2011). A new KSVM+ KFD model for improved classification and face recognition. J. Multimed..

[40] Yang M.H. Kernel eigenfaces vs. kernel fisherfaces: Face recognition using kernel methods. Proceedings of the 2013 10th IEEE International Conference and Workshops on Automatic Face and Gesture Recognition (FG).

[41] Balasubramanian V.N., Ye J., Panchanathan S. Biased manifold embedding: A framework for person-independent head pose estimation. Proceedings of the 2007 CVPR’07 IEEE Conference on Computer Vision and Pattern Recognition.

[42] Chang Y., Hu C., Feris R., Turk M. (2006). Manifold based analysis of facial expression. Image Vis. Comput..

[43] Zhang T., Yang J., Zhao D., Ge X. (2007). Linear local tangent space alignment and application to face recognition. Neurocomputing.

[44] Lin T., Zha H., Lee S.U. (2006). Riemannian manifold learning for nonlinear dimensionality reduction. Computer Vision—ECCV 2006.

[45] Pan Y., Ge S.S., Al Mamun A. (2009). Weighted locally linear embedding for dimension reduction. Pattern Recognit..

[46] Samir C., Srivastava A., Daoudi M. (2006). Three-dimensional face recognition using shapes of facial curves. IEEE Trans. Pattern Anal. Mach. Intell..

[47] Raytchev B., Yoda I., Sakaue K. Head pose estimation by nonlinear manifold learning. Proceedings of the ICPR 2004 17th International Conference on Pattern Recognition.

[48] Fischler M.A., Bolles R.C. (1981). Random sample consensus: A paradigm for model fitting with applications to image analysis and automated cartography. Commun. ACM.

[49] Fiedler M. (1973). Algebraic connectivity of graphs. Czechoslov. Math. J..

[50] Donath W.E., Hoffman A.J. (1973). Lower bounds for the partitioning of graphs. IBM J. Res. Dev..

[51] Phillips P.J., Flynn P.J., Scruggs T., Bowyer K.W., Chang J., Hoffman K., Marques J., Min J., Worek W. Overview of the face recognition grand challenge. Proceedings of the CVPR 2005. IEEE Computer Society Conference on Computer Vision and pattern Recognition.

[52] Vijayan V., Bowyer K., Flynn P. 3D twins and expression challenge. Proceedings of the 2011 IEEE International Conference on Computer Vision Workshops (ICCV Workshops).

[53] Savran A., Alyüz N., Dibeklioğlu H., Çeliktutan O., Gökberk B., Sankur B., Akarun L. (2008). Bosphorus database for 3D face analysis. Proceedings of the European Workshop on Biometrics and Identity Management.

[54] Ouamane A., Chouchane A., Boutellaa E., Belahcene M., Bourennane S., Hadid A. (2017). Efficient tensor-based 2d+ 3d face verification. IEEE Trans. Inf. Forensics Secur..

[55] Kim D., Hernandez M., Choi J., Medioni G. Deep 3D face identification. Proceedings of the 2017 IEEE International Joint Conference on Biometrics (IJCB) IEEE.

[56] Tuan Tran A., Hassner T., Masi I., Medioni G. Regressing robust and discriminative 3D morphable models with a very deep neural network. Proceedings of the IEEE Conference on Computer Vision and Pattern Recognition.

[57] Li H., Huang D., Morvan J.M., Wang Y., Chen L. (2015). Towards 3D face recognition in the real: A registration-free approach using fine-grained matching of 3D keypoint descriptors. Int. J. Comput. Vis..

